# A multisource database tracking the impact of the COVID-19 pandemic on the communities of Boston, MA, USA

**DOI:** 10.1038/s41597-022-01378-3

**Published:** 2022-06-20

**Authors:** Alina Ristea, Riley Tucker, Shunan You, Mehrnaz Amiri, Nicholas Beauchamp, Edgar Castro, Qiliang Chen, Alexandra Ciomek, Bidisha Das, Justin de Benedictis-Kessner, Sage Gibbons, Forrest Hangen, Barrett Montgomery, Petros Papadopoulos, Cordula Robinson, Saina Sheini, Michael Shields, Xin Shu, Michael Wood, Babak Heydari, Dan O’Brien

**Affiliations:** 1 School of Public Policy and Urban Affairs, Northeastern University, Boston, MA 02115 United States; 2 Boston Area Research Initiative, Northeastern University, Boston, MA 02115 United States; 3 School of Criminology and Criminal Justice, Northeastern University, Boston, MA 02115 United States; 4 Department of Sociology and Anthropology, Northeastern University, Boston, MA 02115 United States; 5 Network Science Institute, College of Social Sciences and Humanities, Northeastern University, Boston, MA 02115 United States; 6 Harvard T.H. Chan School of Public Health, Boston, MA 02115 United States; 7 College of Engineering, Northeastern University, Boston, MA 02115 United States; 8 Department of Sociology, Harvard University, Cambridge, MA 02138 United States; 9 Kennedy School of Government, Harvard University, Cambridge, MA 02138 United States; 10 Department of Epidemiology and Biostatistics, Michigan State University, East Lansing, MI 48824 United States; 11 Kostas Research Institute, Northeastern University, Burlington, MA 01803 United States

**Keywords:** Social sciences, Geography, Society

## Abstract

A pandemic, like other disasters, changes how systems work. In order to support research on how the COVID-19 pandemic impacted the dynamics of a single metropolitan area and the communities therein, we developed and made publicly available a “data-support system” for the city of Boston. We actively gathered data from multiple administrative (e.g., 911 and 311 dispatches, building permits) and internet sources (e.g., Yelp, Craigslist), capturing aspects of housing and land use, crime and disorder, and commercial activity and institutions. All the data were linked spatially through BARI’s Geographical Infrastructure, enabling conjoint analysis. We curated the base records and aggregated them to construct ecometric measures (i.e., descriptors of a place) at various geographic scales, all of which were also published as part of the database. The datasets were published in an open repository, each accompanied by a detailed documentation of methods and variables. We anticipate updating the database annually to maintain the tracking of the records and associated measures.

## Abstract (summary)

**Table:** 

Measurement(s)	Neighborhood context
Technology Type(s)	Naturally-occurring data
Sample Characteristic - Environment	City neighborhoods
Sample Characteristic - Location	Boston, MA, USA

## Background & Summary

COVID-19 arrived in the United States in the first half of March, 2020. Over the weeks that followed it became clear that the societal impacts went far beyond the virus itself. They included unprecedented challenges for the economy and labour market^1^, mental health^2^, primary and secondary education^3^, public transit^4^, law enforcement^5^, and housing^6^, among others. Throughout, there was the persistent concern of racial and socioeconomic equity, as the burdens of both the virus and the extenuating systemic disruptions landed disproportionately on communities of colour^7–11^.

Though the themes of the pandemic were present across the country, they were inherently local, being experienced and managed at the municipal and even the neighbourhood level. Boston, MA was one of the metropolises that quickly shut down in March and hardest hit during the first wave of the pandemic; retrospective modelling in fact has indicated that community spread had already been occurring in Boston for weeks^12^. The Boston Area Research Initiative (BARI), a centre dedicated to the use of data and technology to advance equity, justice, and democracy in local communities, committed itself to developing a “data-support system for the city during a pandemic.” This entailed gathering data from multiple administrative and internet sources that together would capture the social, behavioural, and economic disruptions created by the pandemic and how they varied across neighbourhoods.

**Fig. 1:**
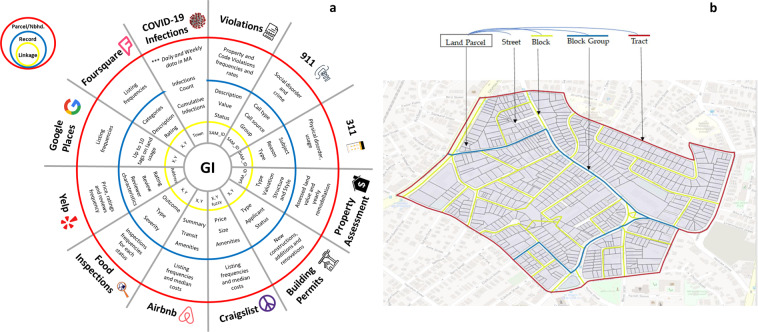
Data-support system for the city during a pandemic: (**a**) Contents of the database, including the associated aggregate measures (red circle), information in the base records (blue circle), and basis for merging with BARI’s Geographical Infrastructure; and (**b**) A visual depiction of the nested schema for BARI’s Geographical Infrastructure.

Here we describe the contents of the data gathered by BARI, including how they were accessed and processed (see Fig. 1a for a list of contents). A critical aspect of the database is that the data come from a variety of sources, and we used BARI’s Geographical Infrastructure^13^ for Boston to spatially coordinate them (see Fig. 1b). This creates two advantages. First, it permits the merger and joint analysis of data sets at multiple geographic scales. Second, all of the data sources comprise records that might reference locations but must be aggregated to create descriptors of those locations. We have developed numerous ecometrics (i.e., descriptors of a space)^14,15^ that use records to describe community features and dynamics (most often at the census tract level). This in turn permits the analysis of cross-community variations in experiences and impacts.

The breadth of the database makes it flexible in supporting numerous substantive questions. We suggest four themes of particular interest, each capturing an important narrative of the pandemic experience in Boston as elsewhere: (1) *Inequitable impacts of the disease*, and the distribution of not only infections but also the extenuating events and disruptions across communities; (2) *Negotiating new norms*, including the public debate around social distancing guidelines and mask-wearing, as well as recalibrating existing systems, like law enforcement and public maintenance; (3) *Economic recovery*, spanning the return of commerce, investment, and growth and how these trajectories vary across communities; and (4) *Building back smarter*, or the way we use these insights in conjunction with an understanding of the wide range of societal disruptions that occurred in order to reestablish systems (e.g., education, public transit) in ways that make them more equitable and resilient. We also provide suggestions of which datasets in the database might be applied to each of these subject areas (see Usage Notes).

The remainder of this Data Descriptor is structured as follows. Methods describes the content and origins of each data set. Data Records provides the file structure and access points for all data. Technical Validation details the steps undertaken to validate the information believed to be contained in each data set as well as the products of the transformation and aggregation processes. Last, Usage Notes provides guidance on linking the data sets and analysing them conjointly as well as other aspects of database maintenance.

## Methods

The COVID in Boston Database contains twelve different datasets drawn from a wide range of sources. This section describes the content and origins of each. We note that, for reasons of data availability and, in certain cases, the timing of developing and deploying web scrapers, the time periods of the data sets are not perfectly contiguous. We publish aggregations annually for full years only and monthly in some cases. We encourage analysts to be mindful of temporal overlaps and disjunctions when using these data and interpreting the results.

In nearly all cases the original records are spatial in nature, referencing locations in the city. For this reason we use the Boston Area Research Initiative’s Geographical Infrastructure (GI)^13^ for the City of Boston, which organizes and links the places and regions of Boston, MA across multiple geographic levels—including land parcels, streets, census geographies, and other administrative regions. The levels are organized in a hierarchy, with the items in each level nested in the higher-level regions that contain it (e.g., land parcels in census geographies). This nesting is coordinated via variables that act as unique identifiers at each level. The records in data sets with spatial information are either spatially joined or geocoded to the appropriate unit of geography. Most often this occurs in either of two ways. First, many of the administrative records maintained by the City of Boston contain one or more identifiers for properties (*PID*) or parcels (*Land_Parcel_ID* which contain one or more properties) or the building lot (*GIS_ID* from the City of Boston’s parcel shapefile, which combines one or more land parcels) drawn from an internal geocoding process known as the Live Street and Address Management (SAM)^16^ system. Alternatively, records that have no such identifiers (e.g., internet-gathered records) but have lat-long information are joined spatially to the nearest building lot (*GIS_ID*).

Connection with the GI’s unique identifiers facilitates two things. First, it enables the creation of aggregate calculations at any geographical scale higher than that at which the data set was mapped (e.g., parcels can be aggregated to street segments, street segments can be aggregated to census tracts, etc.). The resultant “ecometrics” (i.e., characteristics of a space)^14,15^ play a central role in translating these diverse, record-level data sets into analysable, interpretable content. Second, the GI permits merger of aggregations from multiple data sets at a shared geographic scale. This enables conjoint analysis of cross-community variation in experiences and impacts documented by different sources. We describe for each data set how the GI is used, if applicable.

In terms of content, our goal is to give an overview of the types of information contained in the data set and its potential value. Individual variables and additional methodological details are summarized in the data documentation published alongside each dataset (see Data Records for links and citations). We organize the data sets into four groupings based on their primary content: housing and land value; crime and disorder; commerce and institutions; and other.

### Housing and land value

#### Property assessments

The City of Boston’s Assessing Department is responsible for determining ownership and physical characteristics for all properties in the city to ensure fair assessment of both taxable and non-taxable property in Boston. It records this information annually, including valuation (e.g., value of land and structure and amount taxes), structure (e.g., number of floors, rooms, year built), land use (e.g., residential vs. commercial), and other details, for all uniquely identifiable properties. They are released through the City of Boston’s open data initiative^17^.

The data span January 2001 – April 2021. Because a parcel may contain one or more properties (e.g., a condo building contains one property for every condo and one for the main lobby, and often one more for parking), these data are at the property level and are linked to the GI for aggregation to the parcel, building lot, and all higher levels. During processing we introduce additional variables to facilitate informed analysis (e.g., a composite measure that indicates the property’s composite energy efficiency index, based on mechanisms for heating and cooling, among other variables). Aggregate measures are generated that describe the properties within a census tract, including the average assessed value of land, as the ratio of total land value divided by the total property area in square feet, or the mean value of the latest year remodelled across properties.

#### Building permits

Building permits are received and reviewed by the Inspectional Services Department (ISD) of the City of Boston, who organizes and releases approved permits as a final data set through the city’s open data portal^18^. The City of Boston requires that property owners apply for permits before performing substantial work on any structures or erecting new structures. Each permit references a particular type of work to be conducted (e.g., gas, plumbing, excavation, demolition), for which reason a single project could require multiple permits. The impacts of these projects are also assessed by the City of Boston’s Assessing Department and reflected in annual assessments (see above).

The original dataset contains 22 variables describing the work to be done and its anticipated valuation. Using the variables that categorize and describe the building permits, we constructed new dummy-coded variables that classify the types of projects that might be reflected by building permits: *new construction* where there was not previously a structure; *demolition* of an existing structure; *addition* to an existing structure; and *renovation* to an existing structure that falls short of an extension.

This dataset comprises the period September 2006 – January 2021. The latitude and longitude are provided in the City of Boston’s original dataset. BARI uses these coordinates and the parcel ID to link the data to the GI. We first aggregate permits to the parcel level, categorizing the project at a parcel in a given year as one of four types (renovation, addition, demolition, or new construction). The projects at individual parcel are then aggregated to census tracts and block groups to describe two patterns of investment and growth: alterations to existing structures (renovations, additions, and demolitions) and new constructions.

#### Craigslist

We ran a Python script daily to scrape listings from the “housing” section of the five Craigslist URLs that cover Massachusetts (boston.craigslist.org, capecod.craigslist.org, southcoast.craigslist.org, westernmass.craigslist.org, and worcester.craigslist.org) for February 12th, 2020 – January 18th, 2021. The scraper runs once every 24 hours and checks for duplicates already in the database to ensure that a listing is not recorded multiple times if it is online for multiple days. We processed the contents of listings to extract 13 variables that provide information about the property listing and meta-information about the post.

Using the location data (i.e., the latitude and longitude) embedded in listings, we spatially joined the location data to the containing census tract. This was necessary because many of the listings lack the geographic precision to be linked to a lower scale geography. A census tract-level dataset was created that describes aggregate features of neighbourhood property listings, including the quantity of listings and their median cost. These variables were calculated for the entire time period and monthly using the timestamp.

#### Airbnb

InsideAirbnb.com^19^ scrapes information about Airbnb rental listings monthly for select regions, including Boston and Cambridge. Here we include these updates from January 2019 – December 2020. InsideAirbnb collects information about all posts that were active on the date data were collected, meaning each monthly release represents a snapshot of what Airbnb activity looked like in that month. InsideAirbnb’s releases include information on the property’s size, amenities, availability calendar, past reviews, host characteristics, and other features included on the website listing, as well as calculated metrics based on these variables. We have processed and combined these monthly data releases to construct a longitudinal dataset.

Using the latitude and longitude included in each listing we spatially joined to the census block in which it was located and then used the GI to create aggregate measures for census tracts. These measures included listing frequency and median rental value. Additionally, we estimated each neighbourhood’s impact on rental value by using a multilevel regression model to calculate the unique effect each census tract had on rental values when controlling for the number of bathrooms and bedrooms at each property. We calculated all measures monthly.

### Crime and Disorder

#### 911 Dispatches

911 records are generated by the City of Boston’s 911 dispatches, or CAD, (i.e., computer-aided dispatch; from herein, referred to as 911) system. BARI receives these records through a collaboration with the Boston Police Department and releases annual ecometrics for census geographies describing levels of social disorder and crime across the city. These data span 2011–2020.

Cases were linked to the GI using a multi-step process. Initial linkage was based on the *SAM_ID* field from SAM system. If no such value was available or linkage failed we used the *x* and *y* coordinates, if available, to spatially join the event locations with the nearest or containing parcel.

We created a series of ecometrics by tabulating substantively related case types, including: public social disorder (e.g., drunkenness), private conflict (e.g., landlord-tenant dispute), public violence (e.g., fight), and prevalence of guns (e.g., shooting). From 2010–2014 we also generated two additional ecometrics on major medical emergencies (e.g., cardiac arrest) and youth health emergencies (e.g., asthma attack). For census blocks, each measure is a count of events of the associated case types, whereas for the larger census block groups and tracts the measures are rates of events per 1,000 residents. These measures were constructed through an iterative process of content and factor analysis at the census block groups using call types from the 911 record.

#### Code and property violations

Code violations are enforced and tracked by the Public Works Department for breaches of State and City sanitary codes. Building and Property Violations are enforced and tracked by the Inspectional Services Department and address issues with housing, health, sanitation, and safety regulations. They are both released through data.boston.gov (code violations^20^; property violations^21^) and we combine them into a single violations data set as they have similar meaning and structure (e.g., numerous shared variables).

The data span January 2010 – December 2020 and were processed by adding dummy variables for property and code violations (i.e., 1 if the record is in a certain category), a new column for the year, and a dummy feature noting if the violations were among those most commonly used by Boston’s Problem Properties Task Force to determine whether to designate a property as a “problem property.”

We joined the violations to the GI in two steps. First, *sam_id* from the SAM system was used to directly link to *GIS_ID*. Second, for records with no such value, we used *X-Y* (longitude-latitude) to spatially join them to the nearest parcel. We calculated two aggregate measures—one for counts of code violations the other for counts of property violations—for parcels and census tracts. These measures are tabulated annually.

#### 311 Requests

Boston’s 311 system^22^, formerly known as the Mayor’s Hotline and Constituent Relationship Management system, allows constituents to directly request services from the city government and communicates the request to the appropriate department. BARI processes the requests and derives measures that describe: the types of cases received by the system; the reporting patterns of individual users; and ecometric measures of neighbourhood characteristics. These data span January 2010 – December 2020.

Additionally, we scraped requests made through the BOS:311 smartphone application, which is one of the four different channels through which 311 requests are submitted. This dataset complements the usual request information by including comments submitted by users and a unique identifier that can be linked to the main 311 database. The data include requests submitted through the app from January 2020 – March 2021.

A special feature of the 311 system is that those requesting City services can create an account that enables them to track their cases, whether they are using BOS:311 or the other channels. BARI analyses the registered accounts and creates a set of variables describing the reporting patterns associated with them, based on the aggregate of all requests associated with an account.

The 311 records were joined to the GI by the *PROPID* field drawn from SAM. Two categories of ecometrics are derived from the 311 records at the level of the census block group^15^. First, we generate two measures of physical disorder: private neglect, which comprises three submeasures of the failure to maintain private property; and public denigration, which comprises two submeasures of manmade incivilities in public spaces (e.g., graffiti). Second, we generate two measures of reporting patterns: engagement, or the tendency to know of and use the system at all; and custodianship, or the tendency to use the system to maintain public spaces and infrastructure.

### Commerce and Institutions

#### Places of interest

This dataset contains information about places of interest (POIs) in Boston, MA that are captured by Google Places, Foursquare, and Boston’s Tax Assessment Database. To provide researchers with a flexible toolkit for understanding how space is utilized, we set out to compile the most comprehensive POI database possible by combining multiple commonly used POI datasets into a single summary file. The purpose of the POI dataset is to provide microspatial information about land usage across Boston for land parcels. Google Places and Foursquare data were collected via API using a Python script. To ensure that data were collected for all places in Boston, we initially collected data from a sample of locations that expanded outside the geographic boundary of the city.

We overlaid the geographic coordinates (latitude-longitude) of each POI onto the city of Boston’s Tax Assessment Shapefile to reduce the sample to only locations within the city and to link each record to the nearest building lot (*GIS_ID*). POIs whose coordinates fell more than 30 meters from the nearest POI were left unjoined given the uncertainty of the location. Across the three datasets we identified 19,600 parcels (20% of parcels) with at least one non-residential usage in at least one of the three data sets, including 7,760 parcels that had a non-residential property type in the Tax Assessments but were not included in the other two data sets.

#### Food inspections

The Health Division of Boston’s Inspectional Services Department ensures that all food establishments in the City of Boston meet relevant sanitary codes and standards, inspecting all businesses serving food at least once a year. Follow-up inspections are performed on high-risk establishments. The records, which are released through the city’s open data initiative^23^, each describe a discrete violation, meaning that a single inspection can generate multiple records. Each record consists of variables describing the business name, location, overall outcome of the inspection, violation type, severity, any fines, and comments of improvement.

The data span January 2010 – May 2021. We link the food inspections with the Active Food Establishment Licenses^24^ by adding a new column in the data, with value “1” if the food establishment is found on the active licenses and “0” if not (i.e., it is not active or it could not be matched).

The data includes the *property_id* field which is used for joining with the Live Street Address Management (SAM) Addresses^25^, similarly to the Code and Property violations, which then enables a merger on *GIS_ID (PARCEL* in the addresses data). For the elements that did not join, we use the field *location* (combining latitude and longitude) for spatial join with the GI.

We created a crosswalk between restaurants in this dataset and those appearing on Yelp (see more on Yelp below), by applying the Levenshtein distance measure on the joined string of the restaurant name and *Land_Parcel_ID*, providing an ensemble new file with a completed list of restaurants with reviews and inspections in one place. Some restaurants in the ensemble file exist in both databases, while others only in one of them. The released connection table is the most accurate join produced by automatic and manual checks. The crosswalk is only available for January 2010 – August 2020.

#### Yelp

We used a Python script to identify all restaurants in Boston ZIP codes through Yelp. Yelp generates a URL for each restaurant from which we then collected restaurant characteristics (e.g., type, price range, average user rating) and all reviews posted for that restaurant, including limited information about the user who posted the review. The resultant dataset contains information about all restaurants in Boston that were on yelp.com from October 13th, 2004 – August 17th, 2020. To georeference the sampled restaurants, we geocoded the address of each restaurant (as stated by Yelp) using here.com and then spatial joined the resultant latitude and longitude to the nearest land parcel.

Following this spatial join, BARI’s Geographic Infrastructure was leveraged to attribute each restaurant to its containing census tract. We then calculated aggregate measures based on restaurant and review characteristics for all census tracts. To facilitate research about how restaurant visitation changed during the COVID pandemic, we have aggregated the count of reviews by month based on post date.

### Other

#### CityScore

The City of Boston tracks its performance and community well-being on multiple metrics compiled in the CityScore portal^26^. These are also released through the city’s open data initiative^27^. The CityScore metrics include EMS incidents, library users, response to requests for public works services on time, and others. Most measures receive daily scores, reflecting activity from the previous weekday, and monthly scores, reflecting a rolling average of activity over the previous month. Some measures only have monthly scores as they are less reliable at granular time scales (e.g., shootings). For each metric, a score less than 1 indicates that performance for that period was below the target, a score greater than 1 indicates performance exceeded the target. These are calculated according to pre-established benchmarks and a variety of logics (e.g., (measure – target)/target). Occasionally, a score cannot be calculated because there were not tasks on which the city could evaluate performance (e.g., if no streetlight outages are reported, it is not relevant to measure whether they were fixed on time). These data have no spatial component and are not linked to the GI. They are included nonetheless as they have clear relevance to other data sets (e.g., 311 reports) and track the dynamics of the city before and during the pandemic. These data span January 2018 – December 2020.

#### Infection rates

The Commonwealth of Massachusetts’ Department of Public Health has released new positive infection counts and cumulative infection counts since the start of the pandemic for each municipality and county in Massachusetts. Our data release span is April 2020 – January 2021. These counts are weekly for municipalities and daily for counties. The data were released in *.doc format for April 14^th^, 2020 – May 20^th^, 2020 and *.pdf format thereafter. We have extracted this content through January 21^st^, 2021 and reorganized it in datasets in a *.csv format to be more accessible for analysis. As of May 27^th^, 2020 DPH began to include additional, more detailed variables, but in our database we have included only the original new and cumulative counts.

Our database includes imputed daily town measures, calculated as follows: (1) we calculated the weekly sum of infected cases per county (from the weekly cases data per municipality); (2) calculated the percentage of cases in each county attributable to each municipality; (3) assumed that the municipality was responsible for the same percentage of daily cases as their weekly rate to impute daily infected cases.

## Data Records

All the datasets contained in the COVID in Boston database have been published through the Boston Area Research Initiative’s Boston Data Portal repository on Harvard Dataverse and are available for download. They are organized as “datasets,” which in Dataverse terminology are data products that have a unique URL and citable DOI and contain one or more data files that can be downloaded individually (DOI and citation for each dataset is noted below). All datasets are accompanied by a variable-by-variable documentation (i.e., codebook) in *.pdf form, which we omit from the more detailed summary of data files that follows. We have also published a guide^28^ to using the full database. We note that the Dataverse includes a Guestbook by which individuals downloading the data can indicate their identity and organization. This helps BARI to better understand whose efforts the data are supporting (i.e., diversity of public, private, and academic institutions and geographic range). These entries are not validated, however, and users of the data can enter as much or as little information as they choose.

### Housing and land value

#### Property assessments

These data have been processed to generate metrics at two analytic levels^29^:

- *PADCross.Record.YEAR.csv* is the base file derived from the tax assessor’s annual release through data.boston.gov, with additional calculated and merged variables, including geographical descriptors from the GI.
- *PADCross.CT.YEAR.csv* contains ecometrics that describe neighbourhoods (i.e., census tracts). This file is also available as a mappable shapefile (*.shp).
- *PADLong.Record.YEAR.csv* contains a series of variables describing properties’ land use and valuation information year-to-year, including a set of calculated variables that describe their longitudinal changes.
- *PADLong.CT.YEAR.csv* tracks change in assessment and land use over time for census geographies. This file is also available as a mappable shapefile (*.shp).

#### Building permits

Building permits approved by the City of Boston have been processed to generate data files at four analytic levels^30^:

- *Permits.Records.Geocoded.csv* is a modified version of the original Building Permits dataset released by the City of Boston, including all variables in the original data as well as those introduced by BARI to facilitate informed analysis. The latter includes geographical descriptors from the GI.
- *Permits.Ecometrics.LP.YEAR.csv* records whether a project was approved for each parcel in the city in each year and categorizes the nature of the project (i.e., renovation, addition, demolition, new construction). This information is organized as a series of annual files that each contain one row for each parcel (e.g., *Permits.Ecometrics.LP.2020.csv*). *Permits.Ecometrics.LP.Longitudinal.csv* is a wide-form longitudinal file that combines all files of the form *Permits.Ecometrics.LP.YEAR.csv*.
- *Permits.Ecometrics CT/CBG.YEAR.csv* are two sets of neighbourhood-level datasets, one for census block groups the other for tracts. These contain ecometrics describing the forms of building occurring in the neighbourhood, including the proportion of parcels with projects in a given year and the nature of these projects. This information is organized as a series of annual files that each contain one row for each parcel (e.g., *Permits.Ecometrics.CT.2020.csv*). These files are also available as a mappable shapefile (*.shp). *Permits.Ecometrics CT/CBG.Longitudinal.csv* is a wide-form longitudinal file that combines all files of the form *Permits.Ecometrics CT/CBG.YEAR.csv*.

#### Craigslist

The Craigslist listings have been processed to generate data files at two analytic levels^31^:

- *CRAIGSLIST.Listings.csv* is the base file of Craigslist rental listings for February 12^th^, 2020 – January 18^th^, 2021, including all content gathered from the website and any information calculated or incorporated by BARI, including geographical descriptors from the GI.
- *CRAIGSLIST.CT.csv* contains ecometrics that describe census tracts (i.e., neighbourhoods) in terms of their rental listings. It includes measures, such as the listing frequency and median rent, calculated by aggregating information from listings. These are calculated both for the entire period of data collection and monthly therein.

#### Airbnb

The listings gathered by InsideAirbnb were processed to generate data files at two analytic levels^32^:

- *AIRBNB.Listing.csv* contains information about the listings as posted by insideairbnb.com, as well as geographical descriptors from the GI incorporated by BARI.
- *AIRBNB.CT.csv* contains ecometrics that describe census tracts in terms of Airbnb listing frequency and price value. These metrics have been generated by aggregating information about Airbnb listings for each census tract.

### Crime and Disorder

#### 911 Dispatches

The 911 dispatches are processed to generate files at two analytic levels. We do not publish the records themselves per our data sharing agreement with Boston Police Department^33^.

- *911 Call Type Description 2010-14.csv* and *911 Call Type Description 2014-20.csv* describe all call types over both time periods of the data, including the overall frequencies.
- We release three different types of ecometric files. First, we release a single longitudinal file that contains counts of dispatches for each call type for each census tract in each year (*911 Frequencies of Call Type by CT and Year.csv*). Second, a series of files of the form *911 Ecometrics CBG/CT/BLK Longitudinal, [Yearly/Monthly].csv* contain ecometrics for census tracts, census block groups, or census blocks derived from 911 call patterns by year and by month. Third, we also release these files in the long format, with each row corresponding to a unique census geography-year-measure. The yearly ecometrics are also available as a mappable shapefile (*.shp).

#### Code and property violations

Code and Property Violations have been processed to generate data files at three analytic levels^34^:

- *Violations.YEARStart_YEAREnd.csv* contains records of all property and code violations for the years indicated in the file name. The data include all variables from the original records and include categorizations and geographical information from the GI introduced by BARI.
- *Violations.YEARStart_YEAREnd.Parcel.csv* contains yearly counts of each violation type (code or property) for each land parcel in the city, with separate counts for each year indicated in the data set name.
- *Violations.YEARStart_YEAREnd.Tract.csv* contains aggregate yearly counts of each violation type (code or property) by census tract (i.e., neighbourhood) as well as rates per 10,000 residents.

#### 311

The database includes four sets of files^35^:

- *311 Cases.csv* is the record of all requests for services in five-year intervals, as indicated in the file name (e.g., _2010_2014).
- *311 Case Types.csv* describes types of requests received.
- *311 Users.csv* describes calling patterns for all registered accounts with the 311 system.
- *311 CBG Ecometrics.csv* and *311 Tract Ecometrics.csv* contain ecometrics for census block groups and tracts, respectively, derived from 311 call patterns.
- *BOS311.csv* includes records available via the BOS:311 application.

### Commerce and Institutions

#### Places of interest

The Places of Interest dataset consists of three data files^36^:

- *GooglePlaces.POI.csv* contains information about the places of interest represented in the Google Places database, including categorizations created by Google and geographical descriptors from the GI.
- *Foursquare.POI.csv* contains information about the places of interest represented in the Foursquare database, including categorizations created by Foursquare and geographical descriptors from the GI.
- *POI.Summary.csv* contains all parcels from the Boston Tax Assessment database that have a non-residential land usage classification according to the city or at least one place of interest from Google Places or Foursquare attributed to it. Simple descriptions of the places of interest associated with each parcel are included.

#### Food Inspections

The Food Inspections dataset has been processed to generate files at two analytic levels^37^:

- *Food.Inspections.Records.csv* contains all violation records from the original food inspections released by the City of Boston excluding Retail Food Shops, as well as geographical information from the GI introduced by BARI.
- *Food.Inspections.Yelp.Restaurant.csv* consists of aggregated information from both food inspection records and Yelp, merged at the restaurant unit level (name changed to *Food.Inspections.Restaurant.csv* for 2021 release).
- *Food.Inspections.CT.csv* includes aggregated violations per year per each Census Tract.

#### Yelp

The scraped Yelp content has been processed to generate data files at three analytic levels^38^:

- *YELP.Reviews.csv* contains the restaurant reviews gathered from yelp.com and any associated information (e.g., select user characteristics).
- *YELP.Restaurants.csv* contains information about restaurants listed on yelp.com. It includes cross-sectional measures about the number of reviews and unique reviewers calculated by aggregating information from reviews and geographical descriptors from the GI.
- *YELP.CT.csv* contains ecometrics that describe census tracts in terms of restaurant visitation and price value of goods. These measures were generated by aggregating information about restaurants across census tracts.

### Other

#### City score

The data are organized in two main datasets containing metrics intended to reflect the performance of city services^39^:

- *City Score_Daily.csv* contains the city score by day.
- *City Score_Month-Year.csv* contains the monthly rolling average city score per month over multiple years.

#### Infection rates

The Infection Rates data set includes two data files^40^:

- *Weekly_Town_COVID19_MA.csv* contains weekly COVID-19 infection cases per town in Massachusetts.
- *Daily_Town_COVID19_MA.csv* contains daily COVID-19 infection cases per town in Massachusetts.

## Technical Validation

All contents of the COVID in Boston Database are gathered from other institutions who generate them as part of their business processes. As such, they may contain errors or biases arising from the data-generation process. We institute four types of validation checks before publishing the data: (1) *face validity* of records content; (2) *confirmation of geographic information*; (3) *cross-time reliability* of key indicators that should presumably be stable over time; and (4) *convergent validity* of aggregate measures. The first set of checks are specific to the content of each individual data source, the second is generalized across data sets, and the third and fourth involve coordinating aggregate measures from across data sets with each other and with demographic measures from the U.S. Census Bureau’s American Community Survey.

### Face validity of records

The data records are derived from official and public sources, but they might still contain errors of various sorts. For each data source we have identified specific variables whose values merit closer scrutiny. This is done through a series of preliminary analyses and visualizations that can reveal unexpected (or clearly erroneous) details of each variable’s distribution, including descriptive statistics for numeric variables (mean, median, mode, maximum, minimum, histograms, etc.) and frequency tables for character variables. In many cases we either directly edit these values or exclude cases for which a more informative value cannot be inferred. These alterations are translated into rule sets that are executed systematically with code. We describe how we do this for each data set.

In Property Assessments the variable for year built (*YR_BUILT*) often has mistakes, with ‘0’ substituted for unknown values in the original records. We replace these with blanks (NAs). Many properties have an assessed value of $0. These are not mistakes but a reflection of having a tax-exempt land use, therefore their assessed tax value is indeed $0. We also incorporated counts of units within each property from the SAM database. The SAM does not include this information for all properties, however, so we imputed for the remaining properties based on land use, assessed value, gross floor area and living area, omitting inexplicably high outliers. More detail on this process is available in the full documentation.

For data sets measuring crime and disorder (911 dispatches, Code and Property Violations, 311 Reports), all measures are based on the tabulation of records matching pre-defined subsets of case types. The names of these case types occasionally change with shifts to the database system. This happened when 911 switched to a completely new system in 2014 and in more incremental ways for 311. For the former, we have included a crosswalk between old and new case type names in the full documentation. For the latter, we identify situations in which “new” case types are actually truncated or typographically-altered versions of existing case types and reset them to the original case type name to maintain consistency throughout the dataset. In situations when a new case type is in fact novel we leave it as is.

The crosswalk between restaurants in the Food Inspections data and those appearing on Yelp required multiple preprocessing steps entailing spatial and string matching, the result being an ensemble new file with a completed list of restaurants with Yelp reviews and Food Inspections at one place. The released connection table is the most accurate join produced by automatic and manual checks.

To filter outlier Craigslist listings with prices that are likely typos (e.g., rent price > $1 M/month) or designed as attention-grabbing spam (e.g., $1/month) we omitted the highest and lowest 0.2% of records before any aggregation calculations following previous work with these data^41^.

To ensure the Place of Interest summary file only contained non-residential places, parcels not referenced by the online sources were removed if they had a residential land-usage according to the property assessment database (as indicated by having a *PTYPE* value below 300).

### Confirmation of geographic information

We link all data sets containing spatial information to the unique identifiers provided by the GI, most often *Land_Parcel_ID* or *GIS_ID*, to facilitate measurement and analysis at the land parcel level or any higher scale of geography (see Methods for more). After doing so, we conduct multiple checks to confirm that the spatial joining process was successful and did not suffer from systematic errors. These checks also can also uncover errors in the source data, thus allowing us to omit non-sensical cases (e.g., restaurants mapping far outside of Boston despite being caught in a ZIP code-based search of Yelp pages).

The first check we conducted, which occurred before spatial joining, was to identify any locations whose latitude-longitude coordinates fell outside of Boston. It was possible for records to fall outside of Boston for two reasons. One class was fully legitimate in that they were just along the border of Boston. These were kept but not spatially joined. Others were removed because it was clear that they ended up in the data erroneously (e.g., the wrong ZIP code was entered into Yelp, causing a location that is actually in another state to fall into our search criteria) or that the latitude and longitude was entered incorrectly but the event must be in or around Boston (e.g., a 911 dispatch). In the former situation, we remove the cases. In the latter, we keep the records but do not include them in geographic calculations.

The next series of checks was of counts of records for parcels, streets, and census blocks, block groups, and tracts. In each case, we examined the locations with the top numbers of cases (ranging from the top 3 to the top 10, depending on the distribution), manually determining if they make sense. For example, the parcels with the largest number of 911 cases are hospitals, homeless shelters, and other major landmarks. The parcel with the highest number of 311 cases is City Hall, as that is where many cases are reported and is the default location when there is none provided. For census geographies, we also examined places with zero cases to confirm that they make sense as well (e.g., a lack of Yelp locations should be in residential neighbourhoods, a lack of 911 dispatches should only occur in places with very little population). This process also gives us an opportunity to spot-check the assignment of cases to places. We also confirm in this process that street segments with no parcels (just under 50% of street segments; mainly short, undeveloped segments, highways, on-ramps, etc.) have few to no records associated with them.

### Cross-Time reliability of indicators

One way to infer validity of metrics is to assess their longitudinal consistency via cross-time reliability across units of measurement (in this case, census tracts). This ensures that there are not unexpected, sudden shifts in values that would potentially be indicative of errors in the data. Cross-time reliability on its own does not guarantee validity (e.g., all measures at all units could be incorrect in a manner that remains consistent across time), but it can contribute to a comprehensive strategy of validation. We conducted two types of cross-time reliability tests here: the intraclass correlation coefficient (i.e., the percentage of variance accounted for by census tracts rather than cross-time variability); and bivariate correlation between consecutive years. The former is more rigorous but vulnerable to lower values if variability within a census tract over time generates values that overlap with the variability in other census tracts, which is very possible given places with similarities in demographics and urban design; or all census tracts experience a shared trend (e.g., annual cycles in the rental market). The bivariate correlation then provides an additional insight into change between consecutive periods of measurement.

Measures of Housing and Land Value included both scraped measures that spanned a single year (Craigslist and Airbnb) and administrative records that went back to 2017 (for property assessments) and 2015 (for building permits). The former were assessed monthly, and the later annually, making the interpretations of the precise numbers somewhat distinct. Listing frequencies across months demonstrated very high consistency for Airbnb (ICC = 0.98, *p* < 0.001), but were somewhat less consistent for Craigslist (ICC = 0.62, *p* < 0.001). The latter probably was a product of annual cycles in the rental market as well as the disturbances of the early pandemic, which created citywide trends that would lower an ICC. A subsequent check of correlation between consecutive periods, though, found a much higher correlation for Craigslist postings (*r* = 0.89, *p* < 0.001). Assessed values showed similar consistency (ICC = 0.64, *p* < 0.001 for residential land value per square foot; ICC = 0.71, *p* < 0.001 for non-residential land value per square foot), as did the quantity of building permits (ICC = 0.96, *p* < 0.001).

Measures of Crime and Disorder were measured for tract-years for 2011–2020, at which scale they featured strong cross-time reliability as well. 911-derived metrics featured ICC’s ranging from 0.74 - 0.86, and 311-derived metrics featured ICC’s ranging from 0.66-0.87 (all *p*-values < 0.001). The lower ICC here was for public denigration, which saw some year-to-year citywide shifts. The consecutive year bivariate correlation was *r* = 0.97. Code violations featured high consistency (ICC = 0.74, *p* < 0.001), but property violations did not (ICC = 0.20, *p* < 0.001). Upon further examination, the latter findings could be explained by two things: there was a dramatic shift in enforcement from 2013–2015 that led to more than double the number of citations issued, thereby diminishing intraclass correlation; and property violations are often issued in large numbers at a single property after an investigation, causing unexpected jumps and drops in certain tracts between years (year-to-year correlation: *r* = 0.62, *p* < 0.001).

Measures of Commerce and Institutions were largely non-longitudinal as POIs were only scraped once. Yelp activity, though, did show considerable cross-time consistency at the tract-month level. Though the intraclass correlation coefficient was moderate (ICC = 0.68, *p* < 0.001), this was likely impacted by seasonal effects or pandemic-related disruptions that created citywide cross-time variation. The month-to-month correlation was much higher (*r* = 0.96, *p* < 0.001).

### Convergent validity of ecometrics

For every dataset we have generated multiple ecometrics that describe neighbourhood context. In all cases, we have developed the measures based on the logic of the intended measure. Researchers have noted that metrics based on naturally occurring data (i.e., administrative records, internet-gathered content) are vulnerable to biases created by the varied tendency of communities to actually use these systems to report or document events and conditions, whether they reference crime and disorder, housing, economic activity, or otherwise. This has been especially demonstrated with 311 reports, inspiring researchers to develop a validated method for diminishing the bias in measures of disorder based on this particular dataset^15^. We use this methodology here for our measures of disorder. For these and other measures, we also pursue convergent validity by running a series of bivariate correlations to assess whether they are correlated with those things that they logically should be. Nonetheless, we encourage analysts to always consider the data-generation process underlying these data when interpreting results.

The bivariate correlations were conducted between measures in the database with similar content, with a set of standard demographic indicators from the American Community Survey’s 2014–2018 five-year estimates, and with a classification of neighbourhoods as primarily residential or not. The demographic indicators were the most recent estimates available at the time of analysis. Although these indicators technically describe neighbourhoods before the pandemic, they exhibit strong cross-time autocorrelation (ICCs = 0.94–0.98 when examining three separate five-year estimates), giving us confidence that the correlations reported here would be similar in 2020.

**Fig. 2:**
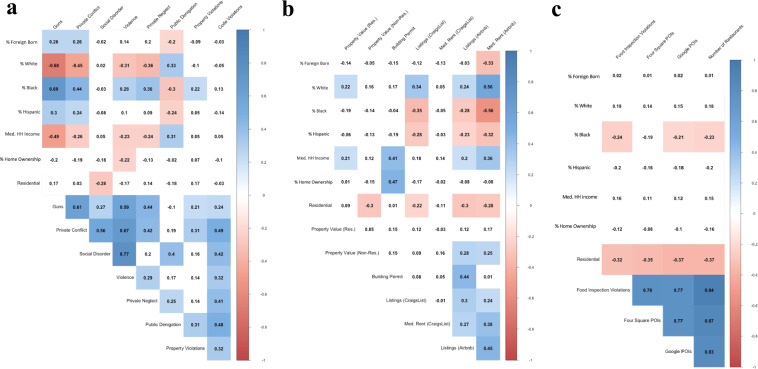
Correlations between ecometrics generated from the multiple datasets and demographic indicators, as relevant to (**a**) housing and land value, (**b**) crime and social disorder, and (**c**) commerce and institutions.

The correlations are reported in a series of correlograms in Fig. 2, organized by class of data (Fig. 2a for Housing and Land Value, Fig. 2b for Crime and Disorder, Fig. 2c for Commerce and Institutions). This acts as a form of convergent validity for determining whether the measures feature the correlations that they presumably should, evaluating both the validity of the aggregation process and the accuracy of the geographic information.

Measures of Housing and Land Value were correlated with median household income and the percentage of White residents, reflecting an expected tendency of greater investment in such neighbourhoods. CraigsList and Airbnb listings and rental values were closely correlated with each other and moderately clustered in regions that are not primarily residential, which is to be expected given the tendency to appeal to tourists and students^42,43^.

Measures of Crime and Disorder were strongly correlated with each other, even when derived from different data sets (see Fig. 2b). They were also higher in lower-income neighbourhoods with higher non-White populations, which is a standard correlation in the criminology literature. The exceptions to these rules were public social disorder (e.g., drunkenness), public denigration (e.g., graffiti), and code violations (closely related to public denigration), which are known to cluster in non-residential areas, thereby leading to deviations from expected demographic relationships. Accordingly, these measures were negatively correlated with tracts being residential.

Measures of Commerce and Institutions unsurprisingly concentrated in non-residential neighbourhoods, which would be expected by definition. They also tended to correlate very highly with each other, reflecting their ostensible effort to catalogue many of the same places. Last, they were substantially negatively correlated with the percentage of Black residents, reflecting lower investment in communities of colour^44^.

## Usage Notes

### Geographical infrastructure

As we have noted repeatedly, all data sets in the database with spatial information are joined spatially with the most appropriate geographic scale of analysis available in BARI’s Geographical Infrastructure for Boston, MA, which consists of seventeen distinct, nested levels for delineating the places and regions of the city (see also Fig. 1b)^13^. Records are most often joined at the level of the parcel (*Land_Parcel_ID* or *GIS_ID*), which is most granular level of analysis at which addresses can be reliably distinguished. The GI enables users to link measures at various geographic scales of analysis and to easily create aggregate measures, based on the nested structure. Studies by the authors have been conducted at the parcel^45^, street^46^, census block^47^, census block group^48^, and census tract levels^49^.

### Updating

The database was originally released in September 2020. It has since been updated through the end of 2020, except for Yelp (owing to manual merging with food inspections) and POIs (under the assumption that they offer background context in many analyses, though future work could consider how the pandemic shifted the institutional landscape of the city). We anticipate updating it annually to maintain the tracking of these various records and associated measures.

### Uses for research and teaching

**Fig. 3:**
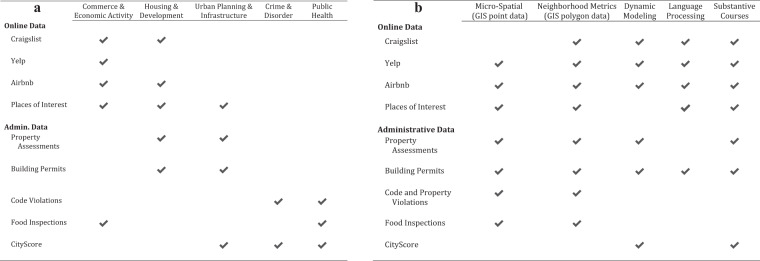
Suggested uses for the datasets in the COVID in Boston dataset, organized by (**a**) substantive narratives of the pandemic, and (**b**) methodological applications.

In the Introduction we noted four main themes for research that capture the narrative of the pandemic in Boston and beyond. Here we elaborate on those opportunities in two ways. First, we have provided a checklist noting whether each data set is relevant to each theme (see Fig. 3a). Second, we offer a few specific suggestions.

1. *Inequitable impacts of the disease*, and the distribution of infections and extenuating events and disruptions across communities is a central theme of the pandemic. It can be probed through any dataset in the database, from the density of COVID-19 infections in a community to shifts in crime and disorder, economic activity, housing, and the provision of government services. For those specifically interested in inequities, places of interest and property assessments offer crucial context, as do indicators provided by the U.S. Census Bureau.
2. *Negotiating new norms*, including the public debate around social distancing guidelines and mask-wearing as well as recalibrating existing systems, like law enforcement and public maintenance, is one of the more striking social phenomena that characterized the pandemic. This is especially apparent in 311 records (which include complaints about non-compliance with COVID-19-instigated restrictions), as well as 911 dispatches (including complaints about parties and fireworks, which increased during the pandemic), and other city systems.
3. *Economic recovery* is one of the most important concerns as society has reopened. Data pertaining to housing, building permits, and economic activity more generally are essential to tracking the return of commerce, investment, and growth and how these trajectories vary across communities, especially when placed in conjunction with demographic data and the persistence of infections.
4. *Building back smarter* demands the deep engagement of researchers with policymakers and practitioners to quantify the impacts of the pandemic, reveal the associated disparities, and suggest and evaluate pathways forward. This is important for re-establishing systems across the wide range of societal disruptions that occurred (e.g., education, public transit), and there are often opportunities to make them more equitable and resilient. For the current database, the opportunity is especially apparent for questions of housing, economic activity through building permits, city services, and sanitation in restaurants. The ability for researchers to use these data to contribute to this process will increase as the data are updated to cover longer and longer timespans.

Last, we designed the database for use in research and teaching. We provide a second checklist of the various methodologies that each data set might support, with the intent of supporting educators to select data sets that might best facilitate the learning goals of a course or module (e.g., network analysis, text mining, spatial analysis; see Fig. 3b).

## Data Availability

All the codes are published through BARI’s GitHub account (user: @BARIBoston; https://github.com/BARIBoston).
